# Practical Notes and Formulæ

**Published:** 1884-07-20

**Authors:** 


### PRACTICAL NOTES AND FORMULÆ.

**Sadberry Spleen Mixture.**—

℞ Quinine sulph … ʒ j,

Ferri sulph … ʒ j,

Acid nit … ʒ j,

Potash nitrate … ʒ iij,

Aquæ … ℥ x.

Teaspoonful three times a day.

℞ Cinchonidia sulph … ʒ j,

Ferri sulph … ʒ j,

Strychnia sulph … grs. ij,

Acid arsen … grs. ij,

Soc. aloes … grs. 10.

M. Ft. pil. 60. One three times a day.

**Remedy for Neuralgic Dysmenorrhœa.**—For neuralgic dysmenorrhœa Prof. Parvin recommends:

℞ Tinct. opii,

Tinct. valerianæ,

Spirit. ætheris comp.,

Tinct. castorei, } aa … f ʒ ij.

M. Sig. A teaspoonful every hour.—*Col. and Clin. Rec.*

**Soldier's Itch.**—If Dr. J. K. Heltman will try the following, he will not have much trouble in curing soldier's itch (prurigo or lichen):

R Sulphur sublimatum … ℥ ij,

Hydrargyri oxydum rubrum … ʒ iss,

Acid boracic … ʒ vi,

Adeps … ℥ viij.

M. Ft. unguent. Sig. Apply every evening.

Before applying the unguent I have them to wash with soft soap, drying with a crash towel. If the crop is not too large one washing will be enough. but sometimes I have them use the soft soap the fifth night again.

As an internal remedy I use the following:

℞ Liquor potassii arsenitis … ʒ ij;

Tinc. cardamom comp … ʒ j,

Elixir simplex … ℥ vj.

M. Sig. Take a teaspoonful after each meal.

I have tried phytolacca, iris versicolor, stillingia and a host of other remedies, but failed to cure until I commenced the above, which has cured in every case where tried.—*Ec. Med. Journal.*

**Remedy for Weakness of the Sexual Organs.**—

℞ Fluid ext. damiana, } aa .................... ℨ iv.

Comp. tinct. cinchona, }

M. Sig. From one-half to one tablespoonful just before or after each meal, and on going to bed at night.—*Med. World.*

**Prevention of Abscess.**—Spread on the fleecy side of Canton flannel, apply to the breast and cover on the outside with oiled silk.

℞ Solid ext. belladonna, } aa ........................ ℨ ij.

Pulv. gum camphor, }

Ungt. resinæ flav ................................ ℥ ij.

M.

**Syrup of Codeia.—For Coughs.**—

℞ Codeia ........................................ gr. j,
Hydrochloric acid, dil ........................ *m* ij,
Distilled water ............................... *m* viij,

Dissolve and add:

Syrup ................................... to .. ℥ j.

Dose, a teaspoonful.—*Ex.*

**Treatment of Infantile Eclampsia.**—The following valuable formulas for the relief of infantile eclampsia during the spasm are published by Wertheimber, of Munchen (in Jahrbuch. fur Kinder-krankheiten.) By preference he employs injections per rectum of hydrate of chloral (0.25 [gr. jv] to aqua dest 50.0 [℥ jss]), since it is more efficient than the inhalations of chloroform. The bromides are quite satisfactory in neuropathic children:

℞ Kali bromat ............................ 2.00 (ℨ ss)

Aquæ dest .............................. 80 0 (℥ ijss)

Syr. simp ............................... 10 (ℨ ijss)

M. D. S. One teaspoonful three to four times daily.

℞ Kali bromat ........................... 2.0 (ℨ ss)

Ammon. bromat ......................... 1.0 (gr. xv)

Aquæ dest ............................ 100.0 (℥ iij)

Syr. simp ............................. 20.0 (ℨ v)

M. D. S. As above.

The combination of the two remedies is very effective.

℞ Kali bromat ...................... 1.5 (gr. xxijss)
Chloral. hydratis ................ 0.5—8 (gr. vijss—xij)
Aq. dest ......................... 100 0 (℥ iij)
Mucil. gum arab .................. 5.0 (ℨj¼)
Syr. cort. aurant ................ 1.50 (℥ss)

M. D. S. Two teaspoonfuls every three to four hours.

In eclampsia due to inanition and collapse, warm baths and excitants, as liq. ammon. anise, etc.—*Ther. Gazette.*

